# Global 3D Imaging of *Yersinia ruckeri* Bacterin Uptake in Rainbow Trout Fry

**DOI:** 10.1371/journal.pone.0117263

**Published:** 2015-02-06

**Authors:** Maki Ohtani, Kasper Rømer Villumsen, Erling Olaf Koppang, Martin Kristian Raida

**Affiliations:** 1 Research Group of Fish Diseases and Immunology, Department of Veterinary Disease Biology, Faculty of Health and Medical Sciences, University of Copenhagen, Frederiksberg, Denmark; 2 Department of Basic Science and Aquatic Medicine, Norwegian School of Veterinary Medicine, Norwegian University of Life Sciences, Oslo, Norway; University of Cape Town, SOUTH AFRICA

## Abstract

*Yersinia ruckeri* is the causative agent of enteric redmouth disease (ERM) in rainbow trout, and the first commercially available fish vaccine was an immersion vaccine against ERM consisting of *Y. ruckeri* bacterin. The ERM immersion vaccine has been successfully used in aquaculture farming of salmonids for more than 35 years. The gills and the gastrointestinal (GI) tract are believed to be the portals of antigen uptake during waterborne vaccination against ERM; however, the actual sites of bacterin uptake are only partly understood. In order to obtain insight into bacterin uptake during waterborne vaccination, optical projection tomography (OPT) together with immunohistochemistry (IHC) was applied to visualize bacterin uptake and processing in whole rainbow trout fry. Visualization by OPT revealed that the bacterin was initially taken up via gill lamellae from within 30 seconds post vaccination. Later, bacterin uptake was detected on other mucosal surfaces such as skin and olfactory bulb from 5 to 30 minutes post vaccination. The GI tract was found to be filled with a complex of bacterin and mucus at 3 hours post vaccination and the bacterin remained in the GI tract for at least 24 hours. Large amounts of bacterin were present in the blood, and an accumulation of bacterin was found in filtering lymphoid organs such as spleen and trunk kidney where the bacterin accumulates 24 hours post vaccination as demonstrated by OPT and IHC. These results suggest that bacterin is taken up via the gill epithelium in the earliest phases of the bath exposure and from the GI tract in the later phase. The bacterin then enters the blood circulatory system, after which it is filtered by spleen and trunk kidney, before finally accumulating in lymphoid organs where adaptive immunity against ERM is likely to develop.

## Introduction

In fish, even formalin-killed *Yersinia ruckeri*, known as a “bacterin”, added to the water as an immersion vaccine can induce immunity against enteric redmouth disease (ERM), caused by *Y*. *ruckeri*. Therefore, this bacterium is ideal for studying the fascinating and fundamental mechanism of antigen uptake in the fish from the surrounding water [1,2]. The first commercially available fish vaccine was an ERM vaccine based on a whole cell *Y*. *ruckeri* bacterin that can be administered to fish by immersion [3–6]. The effect of the ERM immersion vaccine has been demonstrated in controlled laboratory efficacy tests [6,7], as well as in a large field test [8]. However, even though the efficacy of ERM immersion vaccination of rainbow trout is well documented, very little is known regarding the uptake of the bacterin leading to specific immunity in rainbow trout [1,9,10]. Immersion times from as little as 5 seconds have been reported to be sufficient for induction of protective immunity in rainbow trout, but most commercial vaccine producers recommend a 30 second immersion time in order to ensure sufficient antigen uptake for development of immunity [5,9,11]. Besides the time of exposure, development of immunity from immersion ERM vaccination in rainbow trout depends on the weight of the trout [12], and significant protection has been obtained in trout fry at 325 mg [13]. The uptake of *Y*. *ruckeri* bacterin induces an increase in transcripts of several pro-inflammatory genes in the spleen of rainbow trout fry and development of adaptive immunity [7]. Recently it was shown that immersion immunization of trout with *Y*. *ruckeri* bacterin led to the production of *Y*. *ruckeri* specific antibodies and protection against exposure to a *Y*. *ruckeri* challenge [6]. Furthermore, passive transfer of serum from immersion vaccinated fish to naïve trout is known to confer immunity [14,15]. The highest level of protection is obtained with transfer of the serum fraction with highest level of specific antibodies indicating that specific antibodies play a protective role against development of ERM disease [15]. The *Y*. *ruckeri* specific antibodies in rainbow trout are secreted from B lymphocytes and plasma cells in the lymphoid organs such as spleen and kidney [16–18].

Although waterborne administration of the bacterin has been shown to induce systemic humoral or mucosal immunity in rainbow trout, it is generally unknown how the bacterin antigens reach the lymphoid organs and activates an adaptive immune response [9,19]. Several research groups have studied this special route of immunization of fish by use of particles, proteins, whole dead bacteria or other antigens since the 1970s [19–25]. The initial study by Amend and Fender demonstrated that the majority of the uptake of bovine serum albumin (BSA) occurred through the lateral line canal, along with a minor uptake of BSA through the gills of rainbow trout [20]. Moore *et al*. confirmed the results by use of BSA coated latex particles, finding the particles in epithelial cells and underlying phagocytes in the skin and gill, while a minority of the particles were recovered from the kidney and spleen [23].

However studies on uptake of whole bacteria demonstrated that the gills are a main portal of entry and uptake by the lateral line canal was not seen [22]. Using electron microscopy, the uptake of *Y*. *ruckeri* bacterin has been demonstrated in the gill epithelial cells, where especially the pavement cells took up both bacterin and *Y*. *ruckeri* O-antigen coated latex particles by endocytosis [24]. The results obtained by Zapata and colleagues were supported by *in vitro* studies of the uptake of *Y*. *ruckeri* bacterin and O-antigen coated beads via the gills of rainbow trout. This study demonstrated that both bacterin and O-antigen coated beads were taken up within 30 seconds, whereas uncoated beads adhered to the epithelium but were not taken up, leading the authors to suggest that the uptake is selective and specific [19]. Interestingly, it has recently been demonstrated that live *Y*. *ruckeri* initially infect rainbow trout through the gills [26], whereas other bacterial pathogens such as *Renibacterium salmoninarum* do not infect rainbow trout gills [27]. The route of *Y*. *ruckeri* bacterin uptake in rainbow trout is still a subject of discussion, and recently, Khimmakthong *et al*. reported that a *Y*. *ruckeri* bacterin was taken up from the lateral line, dorsal fin, epidermis and gastrointestinal (GI) tract [25].

Knowledge about antigen distribution in organs after vaccination is necessary for our understanding of the mechanisms behind the development of immunity. After immersion vaccination, several antigens including particles, bacterins and soluble proteins have been found to accumulate in the spleen, kidney and liver [21,28,29]. Previous studies on antigen uptake have mainly relied on histological methods. A novel method called optical projection tomography (OPT) has recently been developed to visualize *in situ* three-dimensional (3D) images of gene expression or proteins [30] or specific cells in whole organs in mice [31] based on specific staining of target structures using specific antibodies in organs or small samples. More recently, the OPT method has been used to discover the infection route of *Y*. *ruckeri* O1 in rainbow trout [26], and in the present study, the OPT technique was applied to visualize the bacterin uptake and distribution in whole rainbow trout fry after bath vaccination with killed whole cells of *Y*. *ruckeri*. The 3D OPT images demonstrate the routes of bacterin uptake as well as how the bacterin reach the internal lymphoid tissues in which adaptive immunity may develop.

## Materials & Methods

### Ethics Statement

The study was licensed by the National Animal Experimentation Board (license nr. 2012/561–147) according to the EU Directive EU 86/609. The rainbow trout were treated in accordance with the Animal Experimentation Act of Denmark, which is in accordance with the Council of Europe Convention ETS 123. This government granted license constitutes the level of approval required to carry out animal experiments at the University of Copenhagen.

### Fish

Rainbow trout were hatched from disinfected eggs and reared under pathogen-free indoor conditions at Aquabaltic Hatchery (Nexø, Denmark) and transferred to the experimental fish facility at the University of Copenhagen (Frederiksberg, Denmark) prior to bath vaccination. The average weight of the fish used in this study was 0.32 ± 0.06 g. The fish were kept at 18°C water temperature in 128 L aquaria with internal biofilters (Eheim biofilters, Germany) and air supply ensuring saturated aeration. Commercial feed pellets (BioMar A/S, Denmark) were hand-fed to the fish once per day.

### Bacterin preparation and vaccination

*Y*. *ruckeri* serotype O1 biotype (BT) 1 strain 392 isolated from diseased rainbow trout [32] was cultured in 1L of Luria Bertani broth for 48 h at 20°C with shaking (100 rpm). The number of colony forming unit (CFU) per ml culture media was quantified by triplicate plating of a ten-fold dilution series of the bacteria on blood agar plates (State Serum Institute, Denmark), as described previously [6,7]. The bacteria were inactivated by incubation with 1% (v/v) formalin for 48 hours in room temperature. The formalin-inactivated bacteria were washed twice by centrifugation (4,000 rpm, 15 min), and suspended in 1L of normal tap water, the same as that used for fish rearing. Triplicates of 0.1 ml of suspension were spread on blood agar plates in order to confirm that all bacteria are completely inactivated.

A total number of 55 rainbow trout were bath-vaccinated in 1L aerated water containing 2×10^8^ CFU/ml *Y*. *ruckeri* O1 biotype BT1 for one hour. After bath vaccination, the fish were transferred to a 10L holding aquarium containing clean aerated tap water.

### Sampling

Prior to the time of vaccination, 11 non-vaccinated fish from the control group were sampled to serve as non-vaccinated controls. After the initiation of the vaccination, 11 vaccinated fish were euthanized by an overdose of MS-222 and sampled at the following time points: 30 seconds, 5 and 30 minutes, 3 and 24 hours post initiation of the vaccination. Of the 11 euthanized fish sampled at each time point, 5 were fixed in 4% paraformaldehyde (PFA) for OPT scanning, 3 were fixed in methanol-Carnoy’s fixative solution [60% (v/v) methanol, 30% (v/v) chloroform, 10% (v/v) glacial acetic acid] and 3 were fixed in 4% formaldehyde for immunohistochemistry (IHC).

### Optical projection tomography

Fish sampled for optical projection tomography were processed whole. In order to obtain complete penetration of the specific antibodies and other reagents throughout the whole fish sampled, the opercula were removed on both sides, and small ventral incisions were made in the abdomen of euthanized fish prior to fixation. The samples were fixed in freshly prepared 4% PFA in phosphate buffered saline (PBS, pH 7.4) for 3 h at 4°C. After washing in PBS, samples were dehydrated in increasing methanol concentration (33%, 66% and 100%) for 15 min per step. In order to quench the auto-fluorescence, samples were incubated in methanol:dimethyl sulfoxide (DMSO):30% H_2_O_2_ (2:1:3) at room temperature for at least 2 days, until the pigment disappeared from skin. After two consecutive washes in 100% methanol for 30 min, samples were kept at −80°C in 100% methanol for at least 1 hour and then kept at room temperature for 1 hour. This freeze/thaw cycle was repeated at least 7 times. The samples were then rehydrated in decreasing concentration of methanol (33%, 66% and 100%) in tris buffered saline (TBS) with Triton X-100 (TBST, 2.5 mM Tris-HCl, 4.5 mM NaCl, 0.01% Triton X-100, pH 7.4) for 15 min per step. Samples were incubated in blocking solution (10% goat serum and 0.01% NaN_3_ in TBST) for 48 h at room temperature, and then incubated with 1:2000 fold diluted purified anti-*Y*. *ruckeri* rabbit IgG in blocking solution containing 5% DMSO for 7 days at room temperature [26]. The samples were then washed with TBST for 2 days and incubated with 1:1000 fold diluted secondary antibodies Alexa Fluor 594 conjugated goat anti-rabbit IgG (A11037, Life technologies) for 7 days. Subsequently, the samples were washed with TBST for 2 days, and then embedded in 1% ultrapure low melting agarose (Life technologies). The embedded agarose blocks were trimmed to an optimum size and dehydrated in 100% methanol for 2 days. Finally, the sample was cleared in BABB solution (benzyl alcohol: benzyl benzoate, 1:2) for 2 days, in order to become transparent. The cleared specimen was mounted on a metal disc with glue for the final scanning procedure. For projection of the samples, a Bioptonic 3001M OPT scanner (Bioptonic Inc., UK) was used, and a series of 800 images were captured per 360° rotation. The Bioptonic 3001M OPT scanner records both transmission and emission of light. In this study, the transmission (white light: full-spectrum transmission light) was used to observe the anatomy of fry, while the emitted light was used to detect the auto-fluorescence of fry or the Alexa Fluor 594 fluorophore. No filter was used for the detection of white light, while GFP and Cy3 filters were used to detect auto-fluorescence and the Alexa Fluor 594 fluorophore emission, respectively. The captured images were reconstructed with NRecon software (Bruker microCT, Belgium) and then arranged as a 3D image with Bioptonic Viewer software (Bruker microCT). Overlaying of the captured images was done using Adobe Photoshop CS6, and artifacts were removed manually. 3D film sequences were made using QuickTime Pro.

We have previously performed OPT on rainbow trout infected with live *Y*. *ruckeri* O1 BT1 [26]. However, for this study we adapted the method for use in whole fish. Therefore, all OPT results presented in this publication are obtained using the protocol described here.

### 3D spatial imaging of whole rainbow trout fry

Three of the fish sampled per time point post vaccination were used to optimize and adopt the OPT protocol to detect inactivated *Y*. *ruckeri* in whole fish. Due to this, only one fish sampled per time point post vaccination was successfully processed with optimized protocol for whole fish specimen and scanned by OPT.

The GFP1 channel was used to visualize the internal organs such as the GI tract, liver and heart. However, due to the high numbers of melanomacrophages present in kidney and spleen tissues, white light was better suited. Thin membrane tissues such as the swim bladder and urinary bladder were not detected by OPT by either white light or UV light.

### Immunohistochemistry

In order for the Carnoy’s fixative to penetrate deep into internal organs quickly, the dorsal part including muscle, anterior fin and posterior fin without vertebra, were removed from each sampled fry before fixation. Samples were fixed in Carnoy’s fixative solution for 3 h at room temperature. The fixed samples were then washed twice in 100% methanol for 30 min, followed by two washes of absolute ethanol for 20 min. Finally, samples were incubated twice in xylene for 15 min before being embedded in paraffin. The paraffin embedded samples were sectioned in 4 μm thick coronal sections.

A sample taken at 24 hours post vaccination fixed with 4% formaldehyde was also included for immunohistochemistry, in order to confirm the accumulation of bacterin in various organs. The sampled fry was transversely sectioned into 7–8 pieces and embedded in paraffin. Sections 1 or 4 μm thickness was produced.

The staining procedure of immunohistochemistry was performed as previously described by Chettri *et al* [33], however, with a few modifications. Briefly, the paraffin on the slide glass was melted in 60°C oven for 15 min before deparaffinization steps and an AEC chromogen solution (Thermo Scientifc, UK) was used as a substrate for the HRP conjugated secondary antibody.

## Results

### 1. Distribution of bacterin in trout fry

**Fig 1:**
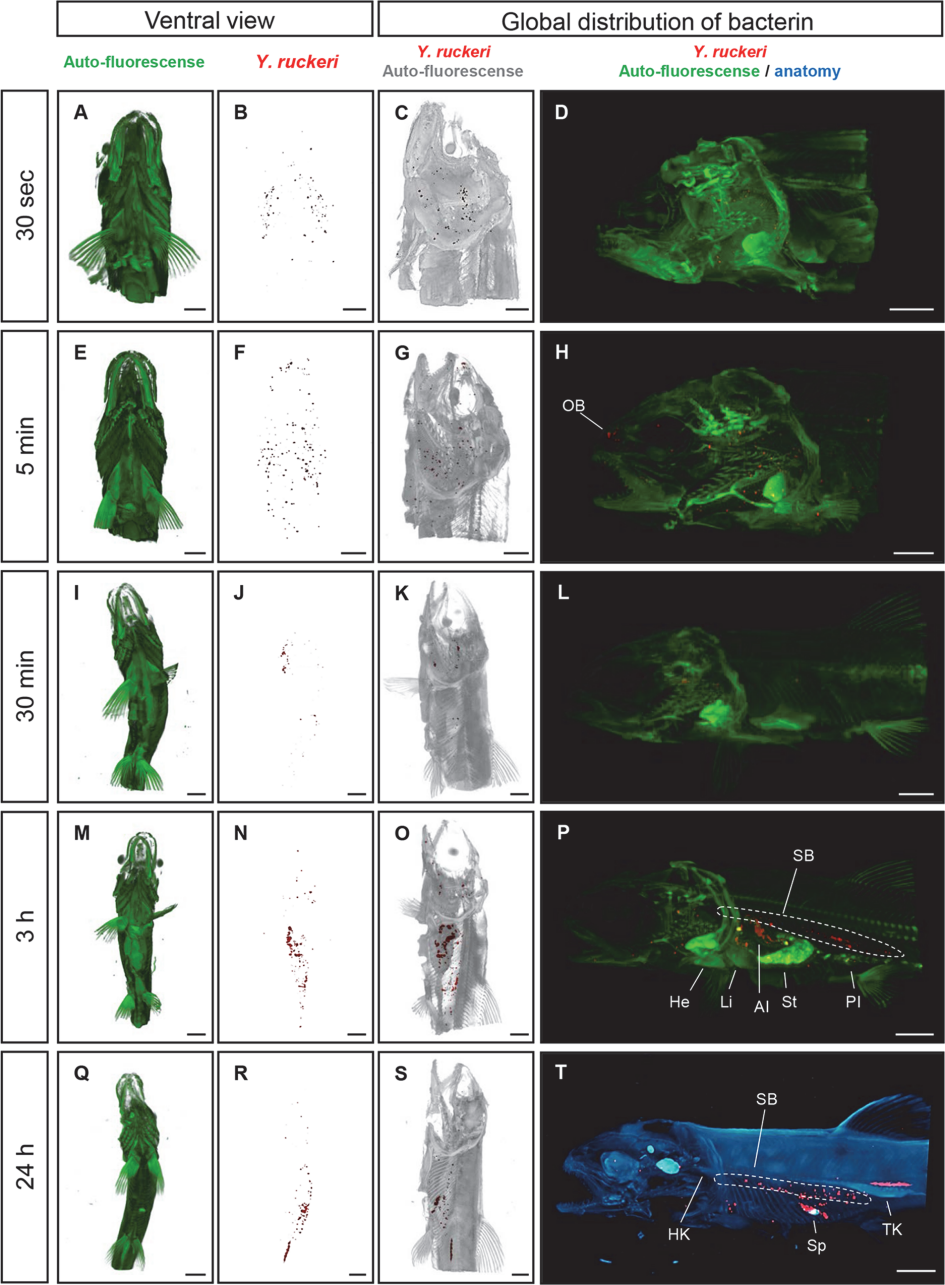
Overview of the 3D spatial distribution of the *Y*. *ruckeri* bacterin over time in rainbow trout fry. (*A*, *E*, *I*, *M* and *Q*) shows a ventral view of the trout fry, the anatomy of the fish is trout based on detection of autofluorescence (green). (*B*, *F*, *J*, *N* and *R*) The red spots showing specifically stained *Y*. *ruckeri* bacterin in the fry shown in A, E, I, M and Q. (*C*, *G*, *K*, *O* and *S*) showing specifically stained *Y*. *ruckeri* bacterin and the gray color is based on autofluorescent showing the trout anatomy. D, H, L, and P are overlaid images of autofluorescent (green) showing the anatomy and the specific stained *Y*. *ruckeri* bacterin (red). The blue color showing the anatomy of the trout based on transmission light and red color demonstrating the distribution of the bacterin. Bar indicates 2 mm. He, heart; HK, head kidney; AI, anterior intestine; Li, liver; OB, olfactory bulb; PI, posterior intestine; SB, swim bladder; St, stomach; Sp, spleen and TK, trunk kidney.

From the OPT scanned samples, the bacterin, in the form of *Y*. *ruckeri*-positive staining, was observed in the gills 30 seconds post vaccination (Fig. 1B‒D). Five minutes post vaccination, positive stains were present in gill lamellae, the olfactory bulb and skin (Fig. 1F‒H), but not in the GI tract or in unvaccinated fish (S1 Fig.). In addition, little amount of bacterin was detected on skin surface of trunk body 30 seconds post vaccination and 5 minutes post vaccination (S2A‒H Fig.). At 30 minutes post vaccination, positive staining was detected in the swim bladder (Fig. 1J‒L). Three hours post vaccination, the GI tract including stomach and intestine as well as swim bladder, showed extensive staining (Fig. 1N‒P, S1 Movie). At 24 hours post vaccination, there were fewer positive stains in the gills, and an accumulation of positive signal was found in the trunk kidney and spleen (Fig. 1R‒T, S3A‒B Fig., S2 Movie). No *Y*. *ruckeri* bacterin in the form of positive signals was seen in any organs of non-vaccinated negative control fish during the experiment (S1 Fig.).

### 2. IHC on transversal section of whole fish

In order to confirm the bacterin distribution indicated by OPT, conventional immunohistochemical staining of whole formalin fixed trout was performed 24 hours post vaccination. The following observations were made:

2.1 Gill

**Fig 2:**
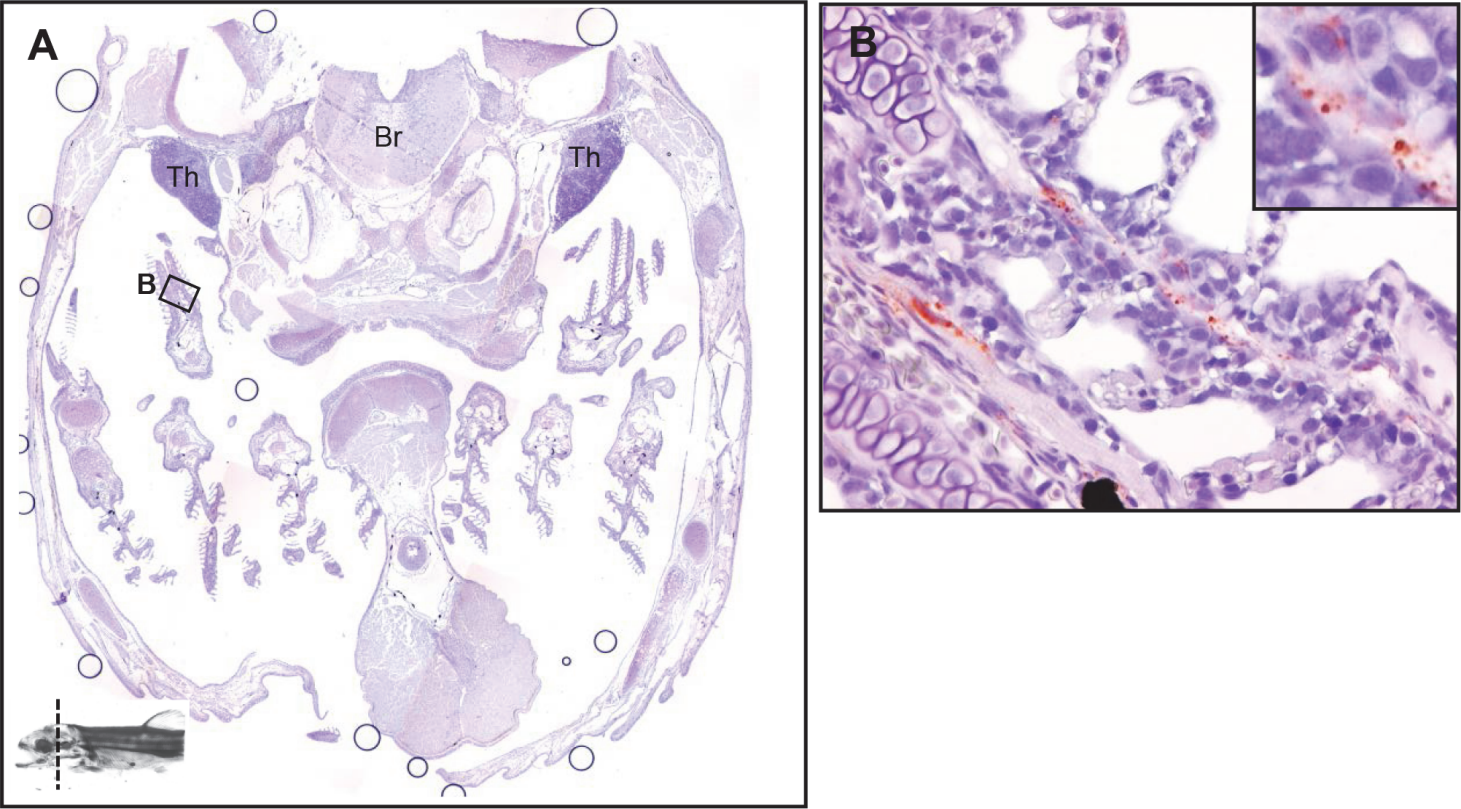
The tissue distribution of *Y*. *ruckeri* bacterin in a rainbow trout fry 24 hours post vaccination (Part 1). A whole fish was fixed in formalin and then sectioned into 5 parts (Fig. 2‒6). The transverse section site is indicated in the bottom of the images on the little fish. (*A*) The head part including gill, thymus (Th) and brain (Br). (*B*) The bacterin is observed in gill capillary.

Compared to the 24h results fixed with Carnoy’s fixative solution, the secondary lamellae showed no *Y*. *ruckeri*-specific signals, most likely since the mucus layer of the gill epithelium is lost during the fixation process, while the positive stains were observed in gill capillaries (Fig. 2B).

2.2 Blood vessels

**Fig 3:**
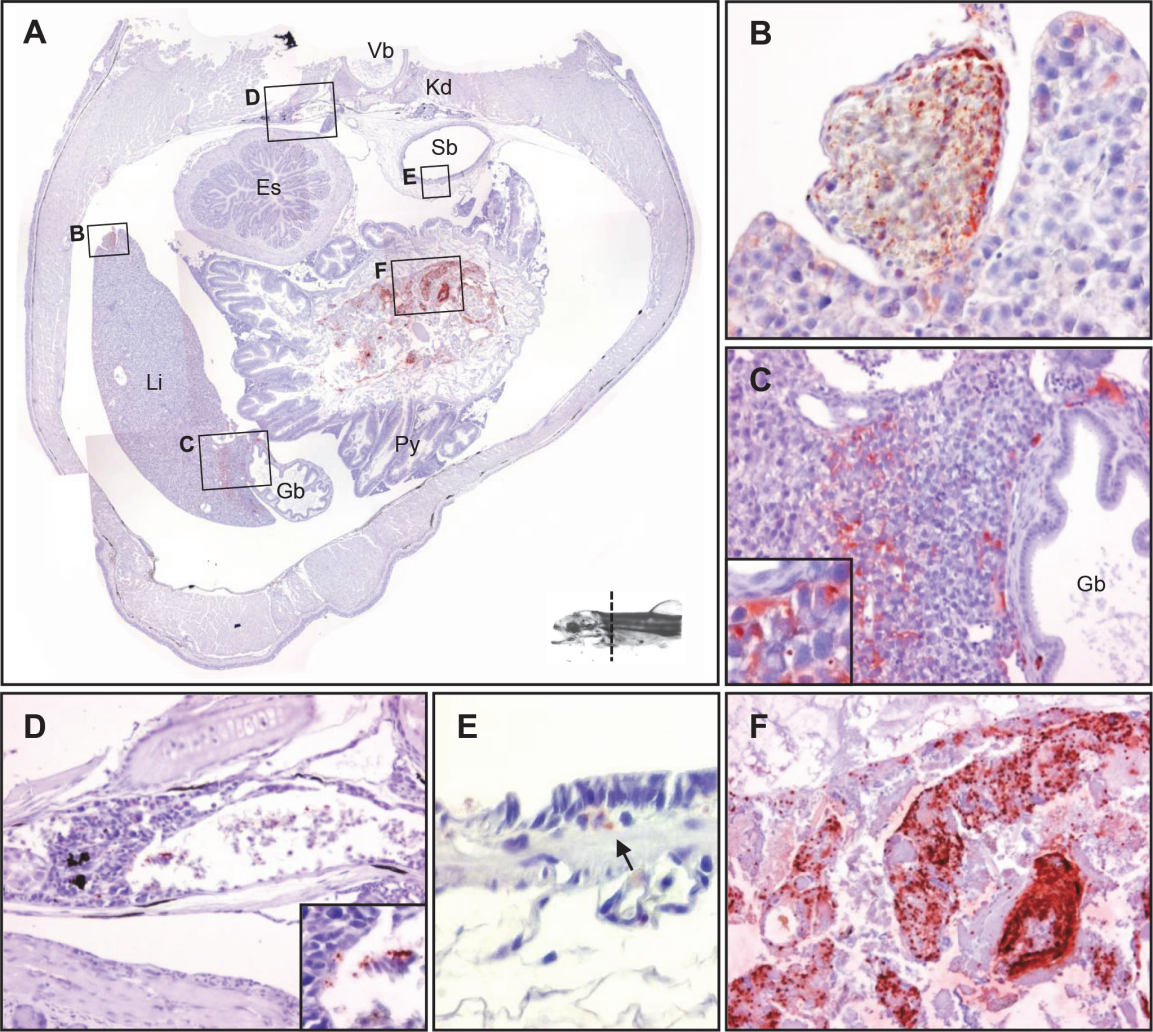
The tissue distribution of *Y*. *ruckeri* bacterin in a rainbow trout fry 24 hours post vaccination (Part 2). (*A*) The section including vertebra (Vb), kidney (Kd), swim bladder (Sb), liver (Li), gall bladder (Gb) and pyloric caeca (Py). (*B*) Blood vessel associated with the liver. (*C*) Liver tissue with gall bladder. (*D*) Kidney. (*E*) Swim bladder. (*F*) Gastrointestinal lumen.

**Fig 4:**
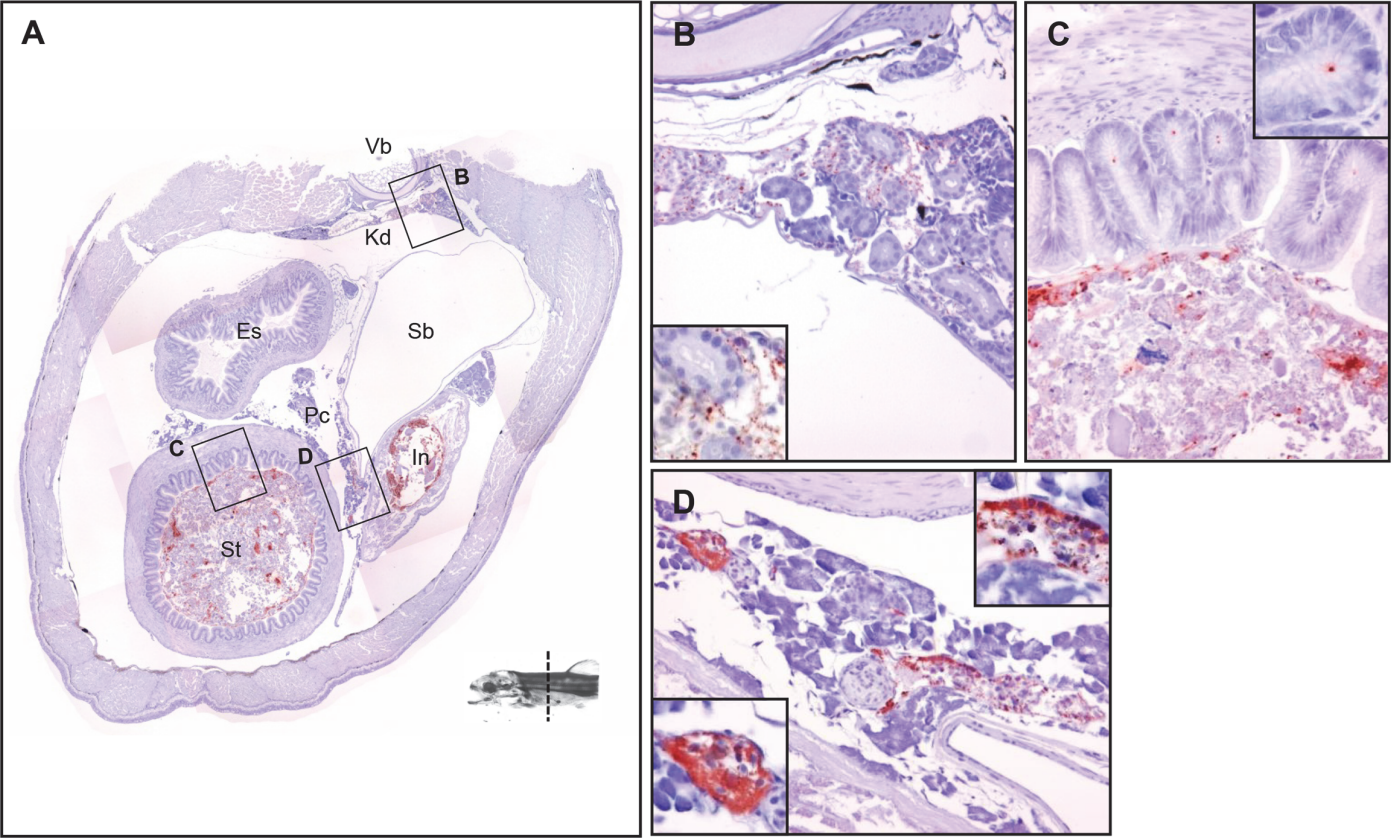
The tissue distribution of *Y*. *ruckeri* bacterin in a rainbow trout fry 24 hours post vaccination (Part 3). (*A*) The section includes vertebra (Vb), kidney (Kd), swim bladder (Sb), stomach (St), intestine (In) and pancreas (Pc). (*B*) Kidney. (*C*) Stomach. (*D*) Pancreas including islet of Langerhans.

Positive stains were observed within the endothelial cells of the blood vessel in the liver (Fig. 3B). An identical scenario was found in the dorsal blood vessel (Fig. 3D). The blood vessels in the pancreas showed widespread positive staining, while no staining was found in the pancreas parenchyma (Fig. 4D).

2.3 Liver

Liver hepatocytes showed an accumulation of positive signals (Fig. 3C). No staining was found in the gallbladder (Fig. 3C).

2.4 Kidney

**Fig 5:**
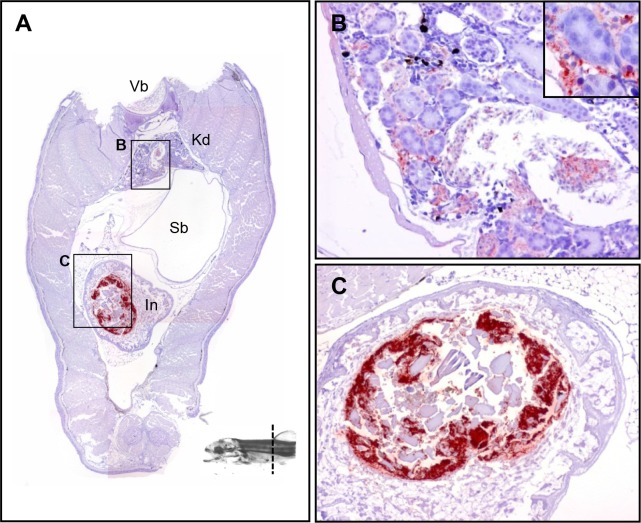
The tissue distribution of *Y*. *ruckeri* bacterin in a rainbow trout fry 24 hours post vaccination (Part 4). (*A*) The section includes vertebra (Vb), kidney (Kd), swim bladder (Sb) and intestine (In). (*B*) Kidney with glomerulus. (*C*) Intestinal content.

**Fig 6:**
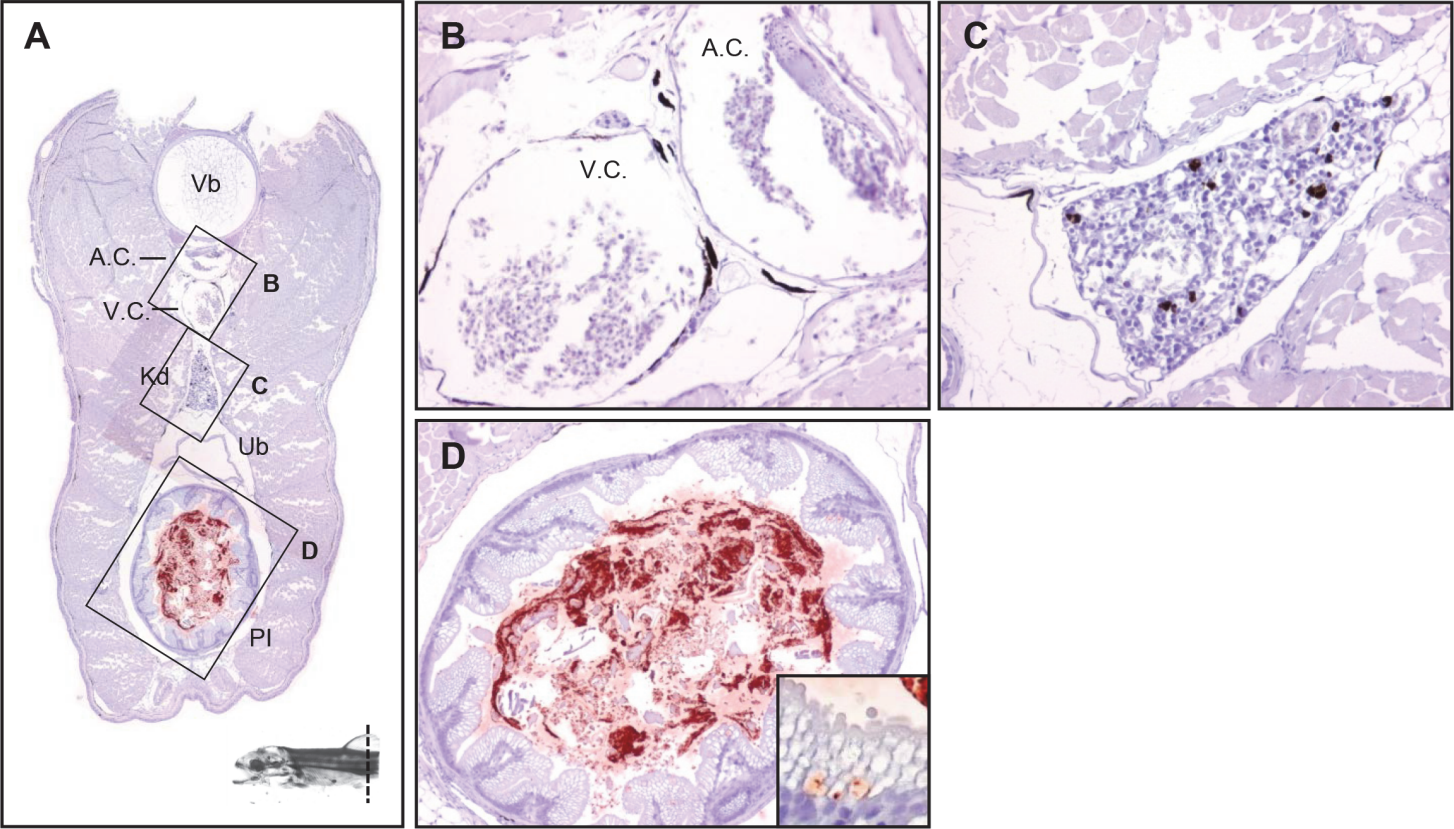
The tissue distribution of *Y*. *ruckeri* bacterin in a rainbow trout fry 24 hours post vaccination (Part 5). (A) The section includes vertebra (Vb), arterie caudalis (A.C.), vena caudalis (V.C.), urinary bladder (Ub) and posterior intestine (PI). (*B*) A.C. and V. C. (*C*) Posterior kidney without nephrons. (D) Posterior intestine.

An accumulation of positive stains was observed in the kidney parenchyma (Fig. 3D). However, more signals were observed in posterior kidney than in anterior kidney (Fig. 4B, 5B). No signals were found in the urethras (the permanent ducts of Wolff) (Fig. 4B and 5B) or at the posterior end of the kidney (Fig. 6C).

2.5 Swim bladder

Positive signals were observed under the swim bladder, but not in the lumen (Fig. 3E).

2.6 Stomach

The lumen appeared to be filled with a mixture of bacterin and mucus, and some identical, positively stained bacterin particles were associated with mucosal surface (Fig. 4C).

2.7 Intestine

The structure of the lamina propria and submucosa in anterior intestine were partially destroyed, most likely by self-digestion during the fixation in 10% formalin. The lumen of the posterior intestine was filled with a mixture of bacterin and mucus, and some identical, positively stained bacterin particles were observed close to the lamina propria (Fig. 6D).

### 3. Immunohistochemistry on fry fixed with Carnoy’s fixative at different time points

In general, little variation in the bacterin uptake was seen between the three replicates sampled per time points from 30 seconds to 3 hours post vaccination, but larger variations were observed in the three fish sampled 24 hours post vaccination.

3.1 Gill

**Fig 7:**
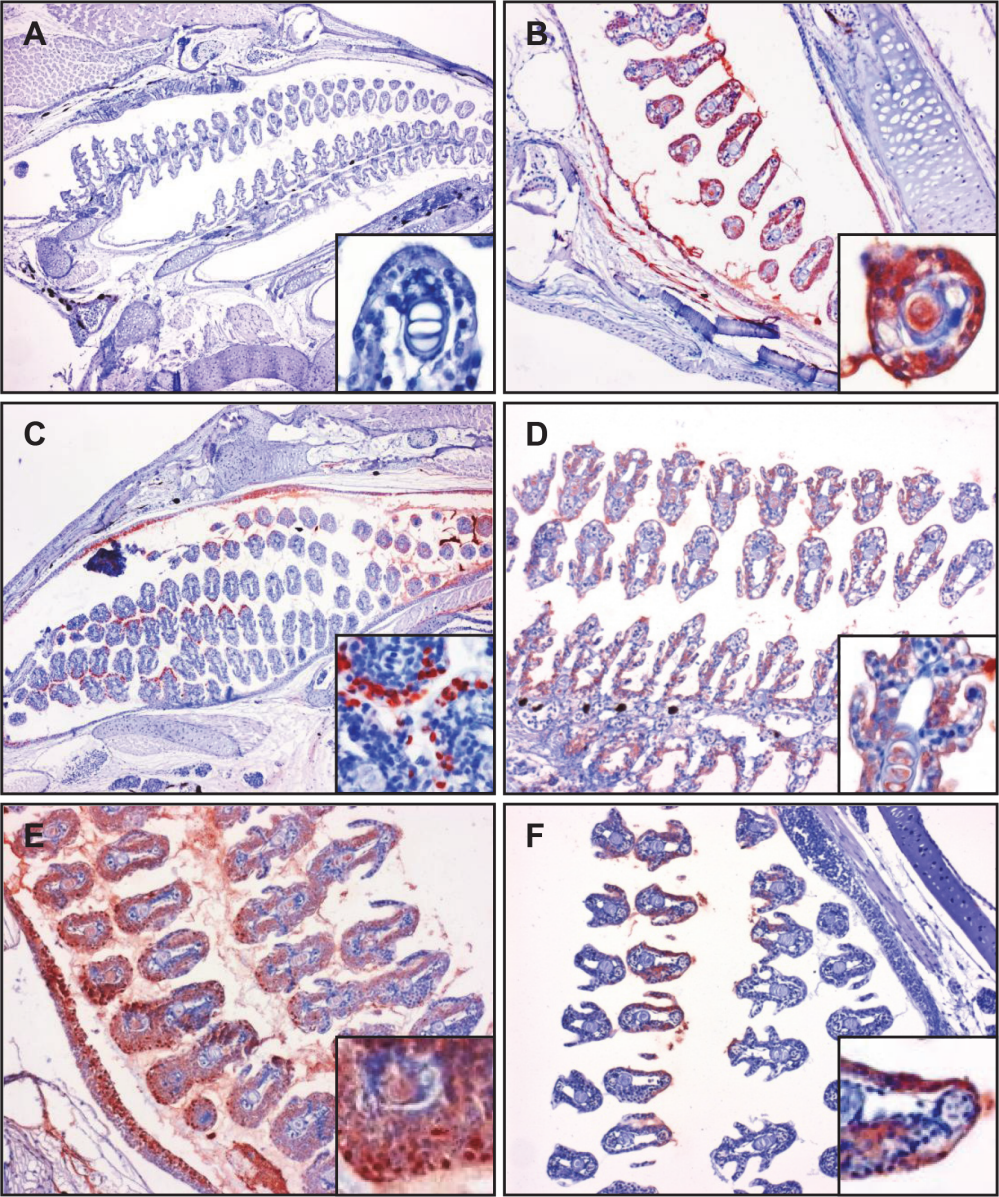
Immunohistochemistry staining of *Y*. *ruckeri* bacterin in gill of vaccinated trout. Gill sections from whole fish fixed in Carnoy’s fixative solution and used for immunostaining of *Y*. *ruckeri* bacterin. The sections are from (*A*) non-vaccinated, (*B*) 30 seconds post vaccination, (*C*) 5 minutes post vaccination, (*D*) 30 minutes post vaccination, (*E*) 3 hours post vaccination and (*F*) 24 hours post vaccination, and were all immunostained with anti-*Y*. *ruckeri* polyclonal antibody as described in materials and methods.

In order to preserve the mucus layer for detection of bacterin attachment to the mucus, three fish per time point were fixed in Carnoy’s fixative solution. Strong staining of bacterin was observed not only within the gill epithelial cells, but also inside the secondary gill lamellae of triplicate samples at 30 seconds post vaccination to 3 hours post vaccination (Fig. 7B‒E). Although the bacterin was continuously detected in gills until 24 hours post vaccination (Fig. 7F), the gills from two of three fish were bacterin negative at 24 hours post vaccination (data not shown). Gill epithelial cells containing *Y*. *ruckeri* in the cytoplasm were observed in specific concentrated areas at 5 minutes post vaccination, while uniformly stained epithelial cells were also observed on same section (Fig. 7C). In addition, the epithelial cells of the gill cavity were also bacterin positive (Fig. 7B, C, E).

3.2 Skin

**Fig 8:**
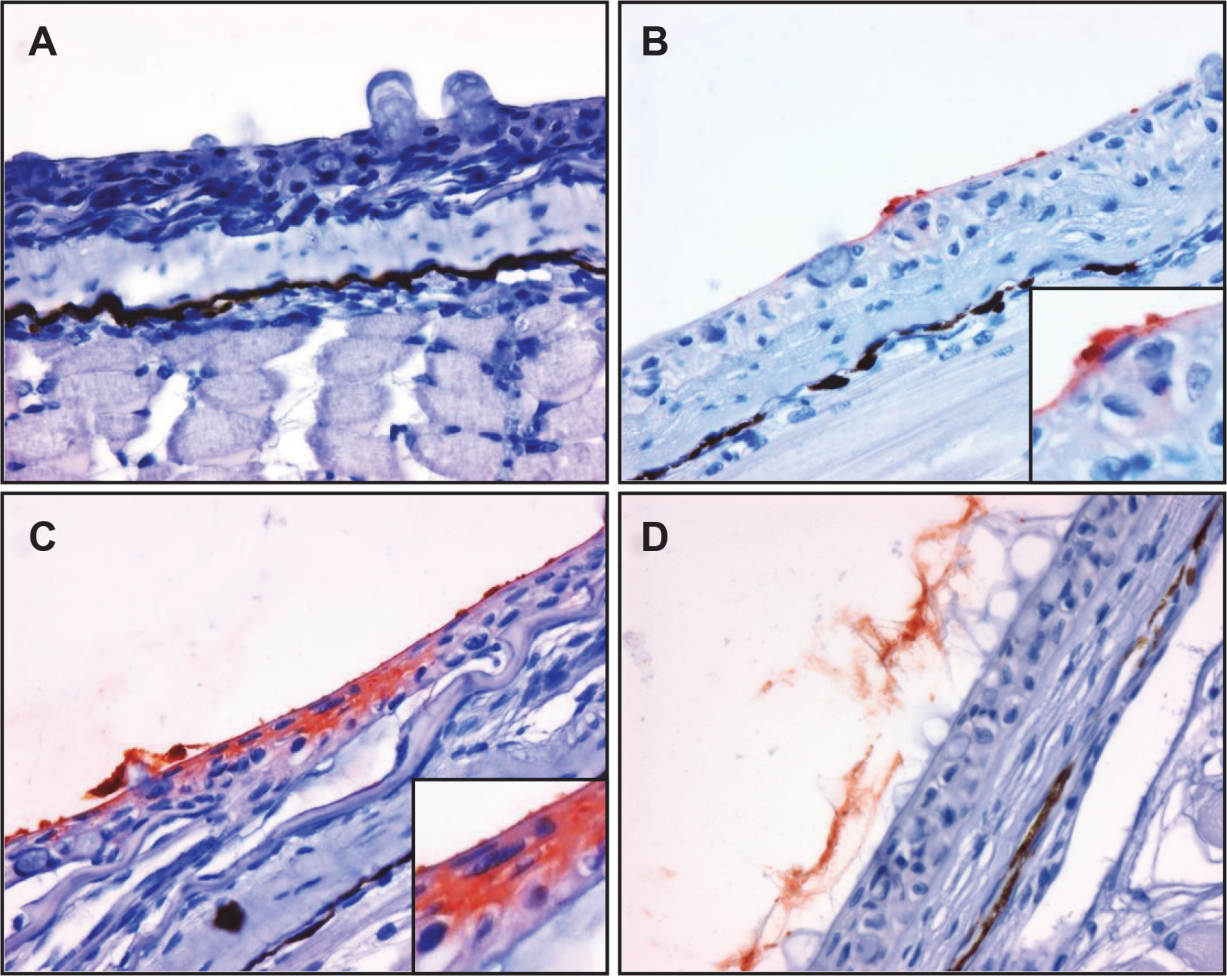
Immunohistochemistry staining showing the association between *Y*. *ruckeri* bacterin and skin epidermis. Skin sections fixed in Carnoy’s solution and used for immunostaining. The sections include (*A*) non-immunized fish, (*B*) 30 seconds post vaccination, (*C*) 5 minutes post vaccination and (*D*) 30 minutes post vaccination. Anti-*Y*. *ruckeri* immunostain. No stain of *Y*. *ruckeri* bacterin was seen in skin samples taken 3 and 24 hours post vaccination (data not shown).

The mucus on the skin surface was successfully fixed in Carnoy’s fixative solution (Fig. 8). The bacterin particles were adhering directly to the skin epithelial surface at 30 seconds post vaccination (Fig. 8B) or trapped by mucus (data not shown). The bacterin components diffused into the epidermis at 5 minutes post vaccination (Fig. 8C). At 30 minutes post vaccination, bacterin associated with mucus was replaced by freshly secreted mucus (Fig. 8D). Adherence of bacterin was not observed in all of fish samples at 3 and 24 hours post vaccination (data not shown).

3.3 Gastrointestinal tracts

**Fig 9:**
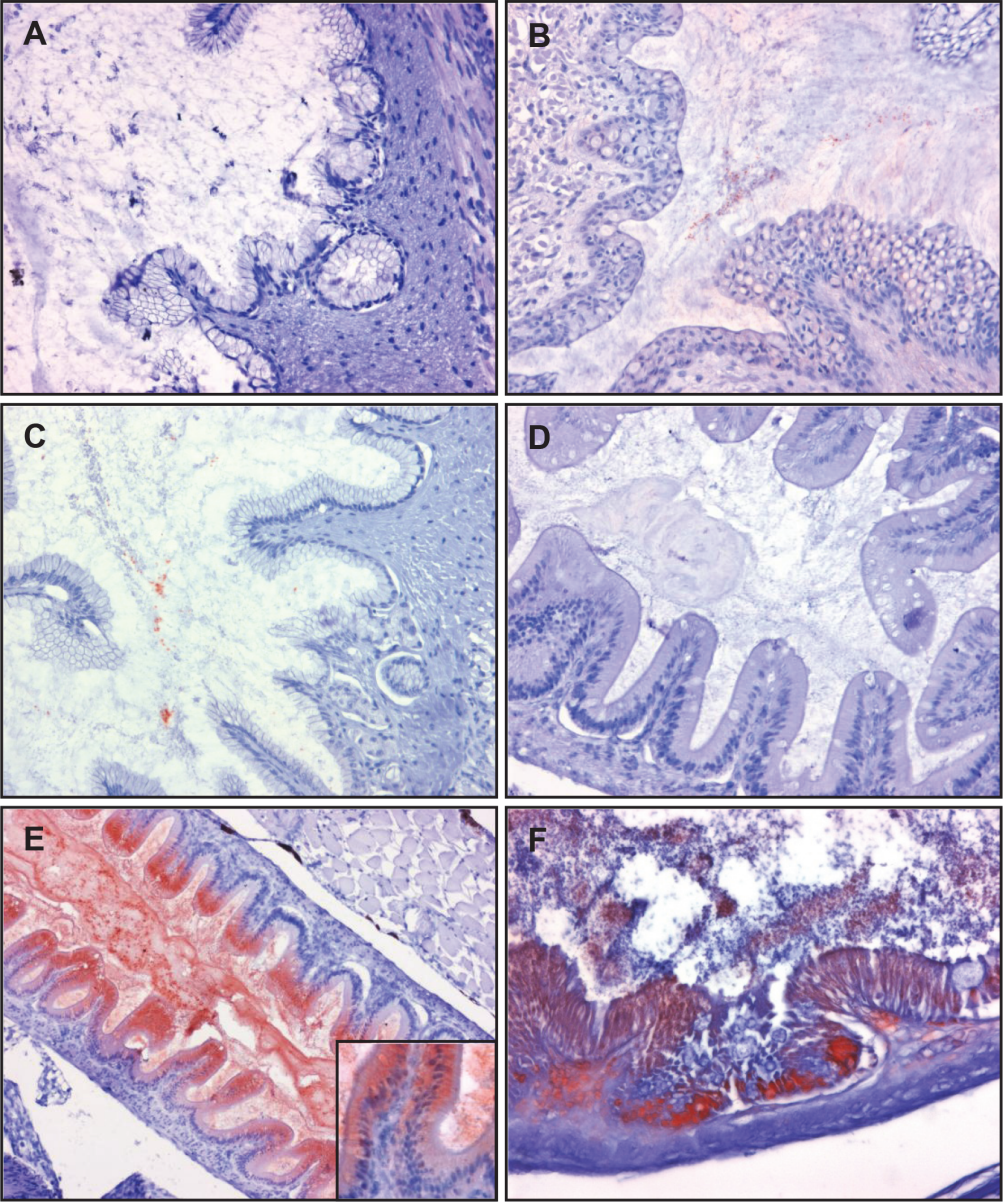
*Y*. *ruckeri* bacterin uptake from GI tract demonstrated by immunohistochemistry. Sections of the GI tract fixed in Carnoy’s solution. (*A*) GI tract of 30 seconds post vaccination, (*B*) esophagus of 5 minutes post vaccination, (*C* and *D*) GI tract and intestine of 30 minutes post vaccination, receptively, (*E* and *F*) intestine of 3 hours post vaccination and 24 hours post vaccination, receptively. Anti-*Y*. *ruckeri* immunostain. Sections are 4 μm.

At 30 seconds post vaccination, the esophagus showed no positive staining in any of the three samples, and in one of three samples, no indication of bacterin was observed in the esophagus until 5 minutes post vaccination (Fig. 9B). Positive stains were observed in all three samples of intestines at both 3 and 24 hours post vaccination (Fig. 9E, F). The bacterin formed a complex with mucus in the lumen, and the enterocytes facing the lumen were bacterin positive 3 hours post vaccination (Fig. 9E). At 24 hours post vaccination, bacterin positive cells were found in lamina propria (Fig. 9F).

3.4 Other mucosal tissues

The bacterin was trapped in various mucosal tissues directly facing the water. In the olfactory bulb, bacterin was detected in one of three samples at 30 seconds (S4A Fig.), two of three samples at 30 minutes post vaccination and one of three samples at 24 hours post vaccination (data not shown). Furthermore, an observation indicated that bacterin was trapped in the mucosal layer of the mouth at 30 minutes post vaccination (S4B Fig.), and also that aggregates of bacterin were attached to the surface of the thymus at 30 seconds post vaccination (S4C Fig.).

## Discussion

Through OPT and IHC analysis of the uptake of *Y*. *ruckeri* bacterin, it was possible to identify plausible portals of uptake, as well as to illustrate the subsequent spread of the bacterin within the vaccinated rainbow trout fry. The credibility of these portals of uptake is underlined by the facts that similar results were obtained using two different immunohistochemical methods to analyze bacterin uptake in several different fish per time point. Bacterin components were initially associated with gill tissues at early time points (30 seconds–5 minutes post vaccination) (Fig. 1). These OPT results were confirmed using IHC (Fig. 7). The OPT results demonstrated that the gills are an initial and probably also the main portal of *Y*. *ruckeri* bacterin uptake during bath vaccination, which corresponds well with previous studies of antigen uptake after immersion vaccination [19,22,24]. Interestingly, the killed *Y*. *ruckeri* bacterin was detected in the vaccinated trout peripheral blood as early as living *Y*. *ruckeri* bacteria are reported to infect rainbow trout [26]. The IHC results showed that bacterin was taken up by gill epithelial cells, but also to a lesser extent by epithelial cells covering the operculum. Nelson *et al*. have previously demonstrated an uptake of *Vibrio anguillarum* bacterin in the operculum cells [29]. Interestingly, particular epithelial cell subsets of the secondary lamellae and opercula were involved in bacterin uptake at 5 minutes post vaccination. Secondary lamella epithelium consists of two cell layers and includes pavement cells and chloride cells [34], of which pavement cell have been proven able to take up bacterin antigens by endocytosis [24]. However, the mechanism of this endocytosis is still unclear.

When studying gill lamellae using a 4% formaldehyde fixation, the mucous dissolves easily due to the large water fraction present during formaldehyde fixation, which poses a risk factor since any bacterin in mucous will then be washed out during formaldehyde fixation. In order to fix the mucous on gill, skin and GI tract, trout fry were fixed in Carnoy’s fixative solution, thus enabling the preservation of the mucus layers of each sample. At 24 hours post vaccination bacterin components were still observed in the mucus covering the gill lamellae, 23 hours after the fry were transferred from bacterin to holding tank with bacterin free water (Fig. 7) considering that mucus would be expected to slough off continuously, the persistence of the bacterin in the gill mucosa is surprising.

The adhesion of live *Y*. *ruckeri* to rainbow trout mucus is mediated by carbohydrates and proteins [35], and therefore it could be suggested that mucus proteins such as mucin might be involved in adhesion of *Y*. *ruckeri* bacterin to mucosal surfaces.

*Y*. *ruckeri* bacterin was observed in skin from 5 to 30 minutes post vaccination in discrete spots along the body of the fry (Fig. 8B) but no bacterin was observed in the skin or the associated mucus 3 hours post vaccination. An uptake of *Y*. *ruckeri* bacterin through the lateral line canal has been described previously but was not observed in this study [25]. Although Smith showed that there are still antigens left on the skin eight hours post immersion into the antigen [21], no *Y*. *ruckeri* bacterin was observed in the skin or the associated mucus after 3 hours in the present study. Ototake *et al* demonstrated that ^125^I-labelled BSA was mainly taken up via skin mucus [36], however as shown by Smith [21] the uptake mechanisms of soluble antigen including BSA and particle antigen such as bacterin or latex bead seems to be different.

Bacterin retention was also observed within the olfactory bulb and in association with the thymus (S4 Fig.). The bacterin was found within the mucous layer of the olfactory bulb. In a similar way, the bacterin on the thymus was just attached on the surface and was not found diffused into the cortex at 30 seconds post vaccination. In a previous study, ^3^H-labelled BSA attached to latex particles was found to have accumulated in thymus at 24 hours post immersion [21].

The salmonid swim bladder is physostomous, connected to the esophagus via a pneumatic duct [37]. Therefore, the bacterin might be transported from the esophagus via water or mucus. In this study, any detection of bacterin in swim bladder using OPT was made impossible whether attached within the mucous layer or not due to the low auto fluorescence of the swim bladder, making it difficult to identify. However, bacterin was observed on the surface or beneath the swim bladder at 24 hours post vaccination (Fig. 3E). In a study by Nelson *et al*. macrophage ingestion of *V*. *anguillarum* bacterin was observed in swim bladder [29], and the newly identified trout dendritic-like cells migrated to the swim bladder in higher percentage than any other organs after intraperitoneal (IP) injection *in vivo* in trout [38], indicating that the swim bladder could have an interesting function regarding antigen uptake from the surrounding environment. In this study, a presence of bacterin in the lumen of the swim bladder could not be detected using immunohistochemistry due to technical limitations of the fixation process. It is necessary to find a way for the fixative solution to penetrate into the swim bladder, which is placed in the center of the body and filled with air.

Recently, the swim bladder was also identified as a site of multiplication for *Y*. *ruckeri* after IP injection or immersion infection [39]. In their study, Mendez and Guijarro observed multiplication of *Y*. *ruckeri* in the swim bladder from 24 hours post infection by IP injection, after which they spread to internal organs. When infected by immersion, the multiplication in swim bladder was observed at 72 hours post infection.

In the present study, the lumen of the intestine was found to be filled with bacterin and enterocytes contained bacterin antigens at 3 hours post vaccination (Fig. 9E). Many researchers have suggested that the GI tract, the second segment of the intestine in particular, is a main portal of antigen uptake [40]. Rombout *et al*. showed that bacterin was taken up from epithelial cells in the intestine and transported by intraepithelial macrophages in carp [41]. Furthermore, McLean and Ash demonstrated a direct uptake of antigen from the lumen by intraepithelial macrophages that subsequently migrated to the kidney or spleen via the systemic circulation in carp [42]. In contrast, Joosten *et al* showed that macrophages containing antigen were not found in the second gut segment in trout even though they were found in the second gut segment in carp [43]. Thus, the mechanism of antigen uptake from the GI tract seems to differ between fish species.

Blood vessels of the pancreas, liver and kidney were found to contain bacterin particles at 24 hours post vaccination (Fig. 3 and 4). In a previous study on blood clearance in fish, MacArthur *et al* demonstrated that 90% of intravenously injected carbon particles were cleared from circulation within 30 min [44]. Therefore, it seems most likely that the bacterin particles in blood at 24 hours post vaccination have not been taken up via the gills, but via the intestinal bacterin content observed at later sampling time points.

Kidney and spleen are major lymphoid organs in teleost containing lymphocytes, antigen presenting cells and antibody producing cells [45]. When in circulation, IP injected antigen has been shown to accumulate in the ellipsoids of the spleen, after which they were transferred to melanomacrophage centers, or transferred to the kidney [46]. Similarly, Smith showed the accumulation of latex beads coated with^3^H-labelled BSA in kidney, spleen, liver and thymus from 24 hours post immersion [21]. Both of these previous observations correspond well with our present results. Furthermore, recent molecular studies have shown that the expression of immune response associated genes were up-regulated in lymphoid organs, such as kidney and spleen after vaccination against *Y*. *ruckeri* [7,47,48]. Therefore the accumulated antigen or bacterin is most likely activating the immune response observed in the lymphoid organs. Much of the bacterin was found in connections with ducts in the kidney and this finding may be explained by increased blood flow to the excretory urine producing part of the kidney which is highly active in fresh water fish [37]. The function of antigen accumulation in the liver is not readily evident from the observations made in this present study. We suggest that the bacterin is taken up in the GI tract and was transported with the blood in the vena porta to the liver. However, soluble antigen such as ^3^H-labeled BSA (without latex bead) or FITC-BSA was not found accumulated in liver [21,36]. These differing observations could be due to different antigen uptake mechanism depending on the antigen type or the size of the fish.

Although the efficacy of immersion vaccination against ERM has been shown by various studies, the nature of the protective immunity has been debated [6,17,18,49–51]. Significantly increased antibody levels of *Y*. *ruckeri* specific circulating antibodies have been demonstrated in ERM bath vaccinated rainbow trout which were also immune to *Y*. *ruckeri* challenge [6]. Recently, passive transfer of serum from bath vaccinated rainbow trout to naïve trout, has demonstrated that it was possible to pass the protection. The author further demonstrated that the protective fraction of serum contain the specific antibodies [15]. Apart from serum IgM, Raida and Buchmann showed that the gene expression of IgT in spleen was increased 10-fold by bath vaccination with *Y*. *ruckeri* bacterin [7]. Zhang *et al*. demonstrated that most gut luminal bacteria were coated with IgT and the number of IgT^+^ B cells was significantly increased after stimulation with *V*. *anguillarum* bacterin in rainbow trout [52]. In the light of the studies mentioned above, it is strongly suggested that specific *Y*. *ruckeri* antibodies in vaccinated rainbow trout could play a vital role in the protection against *Y*. *ruckeri* infection.

**Fig 10:**
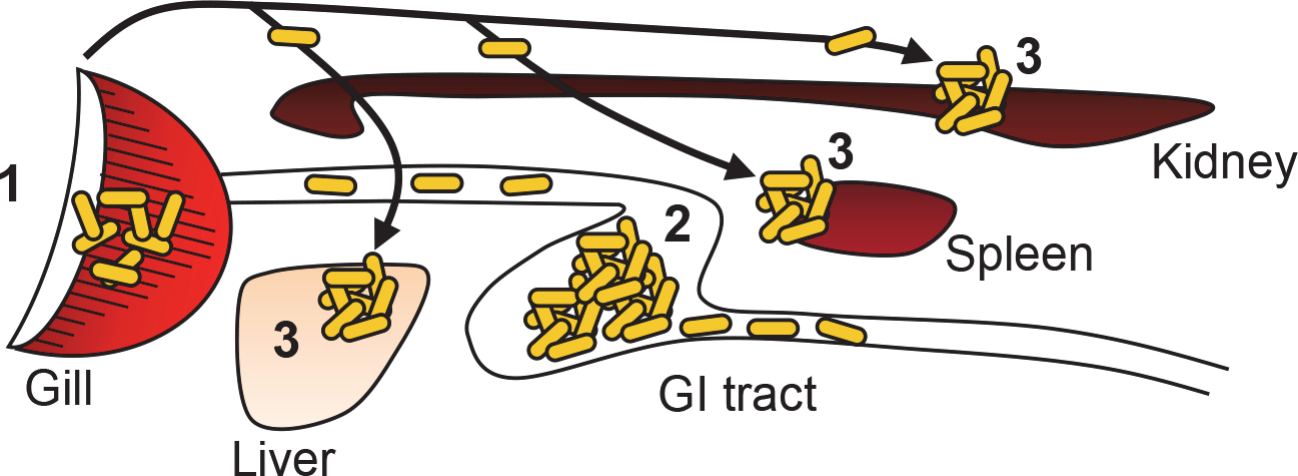
Sequences of *Y*. *ruckeri* bacterin detection in organs. Yellow ovals indicate *Y*. *ruckeri* bacterin. Numbers (1−3) are showing when *Y*. *ruckeri* bacterin detected in the present study as following; 1, 30 seconds post vaccination: 2, 3 hours post vaccination: 3, 24 hours post vaccination. Solid lines show blood circulation system.

In conclusion, the present results suggest that the gills serve as a major point of bacterin entry during the actual immersion vaccination (Fig. 10). After the actual immersion, a buildup of ingested bacterin is suggested to be taken up via the intestine over the next 24 hours, at least. The trapped bacterin is then accumulated in lymphoid organs such as kidney and spleen where adaptive immunity develops and specific antibody producing B- and plasma cells are found. In addition to these conclusions, OPT was found to be a strong tool for tracing the bacterin uptake in whole fish during and following vaccination.

## Supporting Information

S1 Fig 3D image of non-vaccinated rainbow trout fry. The auto-fluorescence was shown with green. There was no staining with polyclonal anti-*Y*. *ruckeri* antibodies (red color).
Bar indicates 2 mm.
(TIF)

S2 Fig The 3D spatial distribution of the *Y*. *ruckeri* bacterin from trunk body in rainbow trout fry. (*A*, *B*, *E* and *F*) The ventral view of trunk part of trout fry, the anatomy of the fish is outlined on detection of autofluorescence (green). (*B* and *F*) Total bacterin uptake in trout shown in A and E. (*C* and *G*) Red spots showing specifically stained *Y*. *ruckeri* bacterin and gray is autofluorescence. (*D* and *H*) Overlaid images of autofluorecsence (green) showing the anatomy and binding of *Y*. *ruckeri* specific antibodies (red). Bars indicate 2 mm.
(TIF)

S3 Fig Association between bacterin and lymphoid organs in 24 hours post vaccinated fish. (*A*) The accumulated bacterin in the spleen. (*B*) The accumulated bacterin in blood vessel.
(TIF)

S4 Fig Detection of *Y*. *ruckeri* bacterin uptake from mucosal and non-mucosal tissues by immunohistochemistry. The sections of the GI tract fixed with Carnoy’s solution. (*A*) Olfactory bulb 30 seconds post vaccination, (*B*) mouth 30 minutes post vaccination, (*C*) thymus 30 seconds post vaccination were immunostained with anti-*Y*. *ruckeri* polyclonal antibody as described in materials and methods.
(TIF)

S1 Movie Global distribution of the *Y*. *ruckeri* bacterin in rainbow trout fry 3 hours post bath vaccination. The anatomical structure and bacterin were shown in green and red, respectively.
(MOV)

S2 Movie Global distribution of *Y*. *ruckeri* bacterin in rainbow trout fry 24 hours post vaccination. The anatomical structure and bacterin were shown in blue and red, respectively.
(MOV)
