# Supplementary figures and images for: Global 3D Imaging of Yersinia ruckeri Bacterin Uptake in Rainbow Trout Fry
Source: PLoS One. 2015 Feb 6;10(2):e0117263. doi: 10.1371/journal.pone.0117263 (PMC4319897; doi:10.1371/journal.pone.0117263)

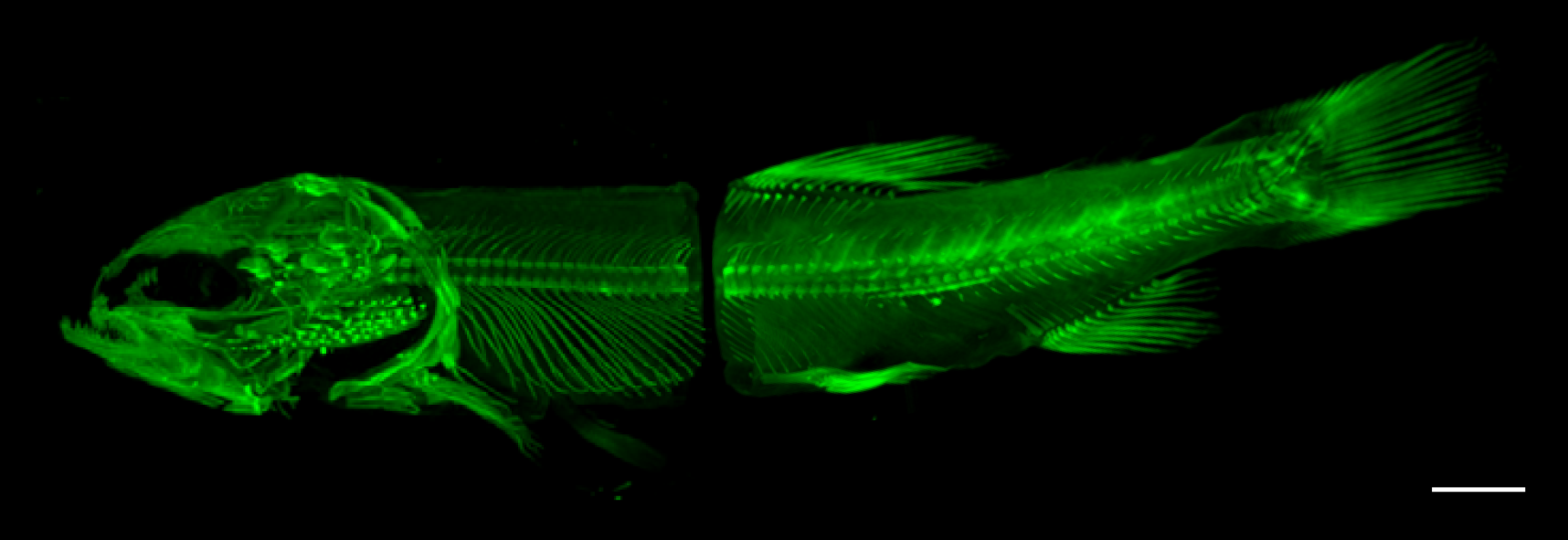

Supplement: S1 Fig — The auto-fluorescence was shown with green. There was no staining with polyclonal anti-Y. ruckeri antibodies (red color). Bar indicates 2 mm. (TIF) [file pone.0117263.s001.tif]

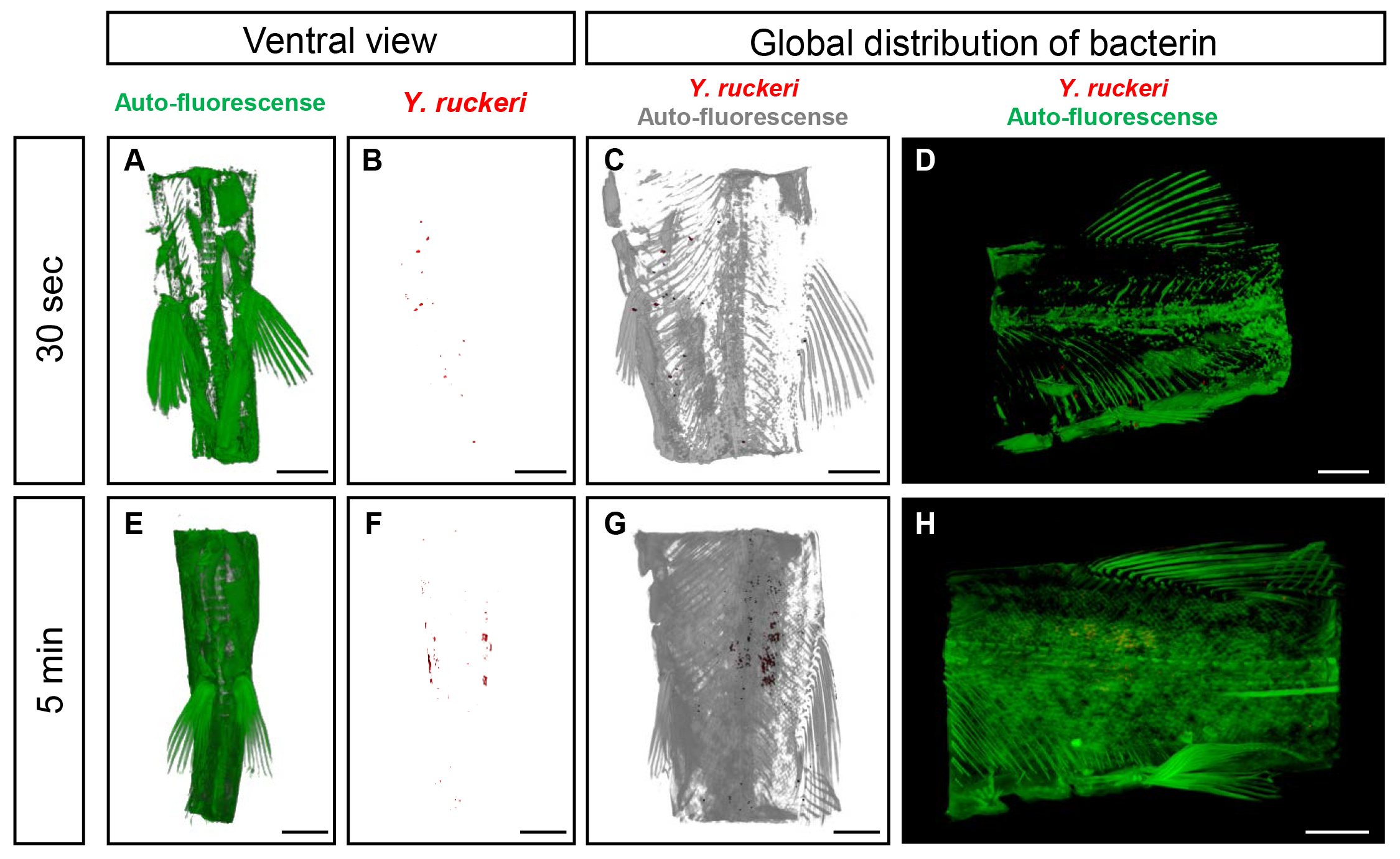

Supplement: S2 Fig — (A, B, E and F) The ventral view of trunk part of trout fry, the anatomy of the fish is outlined on detection of autofluorescence (green). (B and F) Total bacterin uptake in trout shown in A and E. (C and G) Red spots showing specifically stained Y. ruckeri bacterin and gray is autofluorescence. (D and H) Overlaid images of autofluorecsence (green) showing the anatomy and binding of Y. ruckeri specific antibodies (red). Bars indicate 2 mm. (TIF) [file pone.0117263.s002.tif]

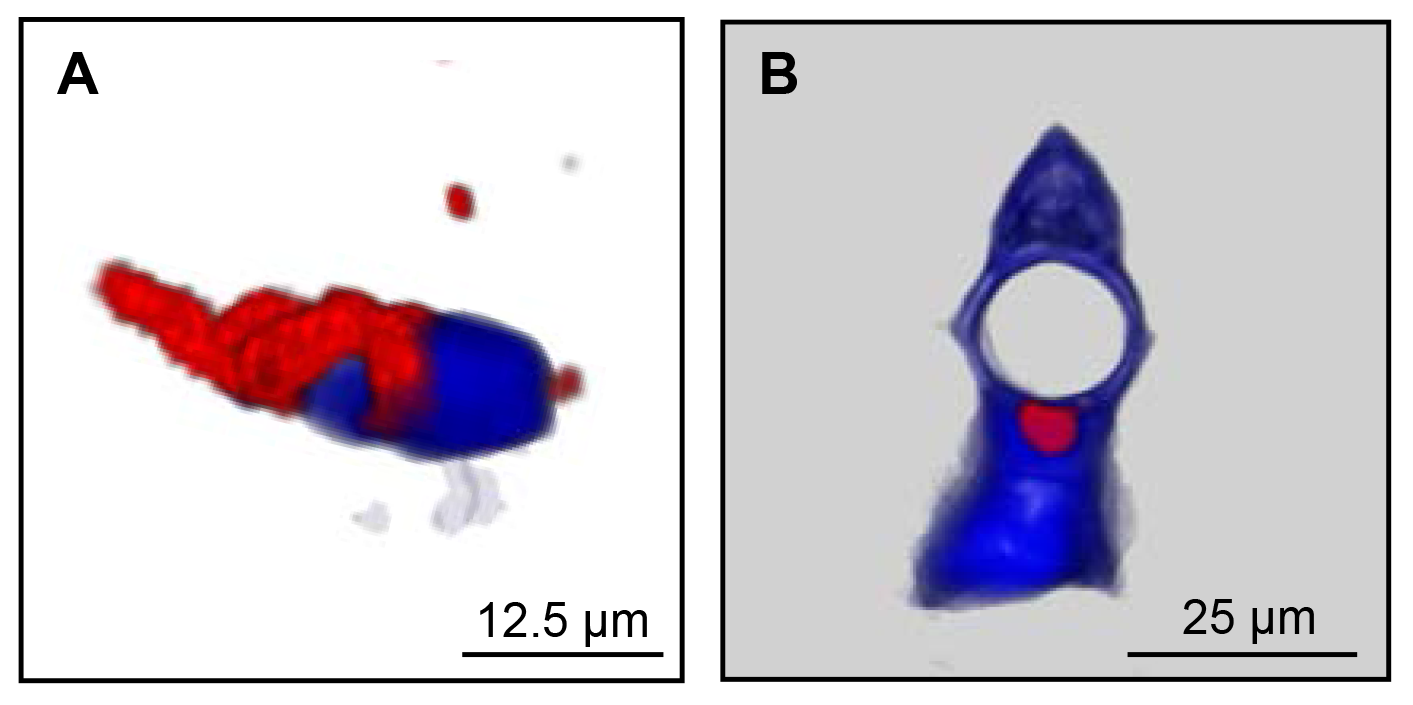

Supplement: S3 Fig — (A) The accumulated bacterin in the spleen. (B) The accumulated bacterin in blood vessel. (TIF) [file pone.0117263.s003.tif]

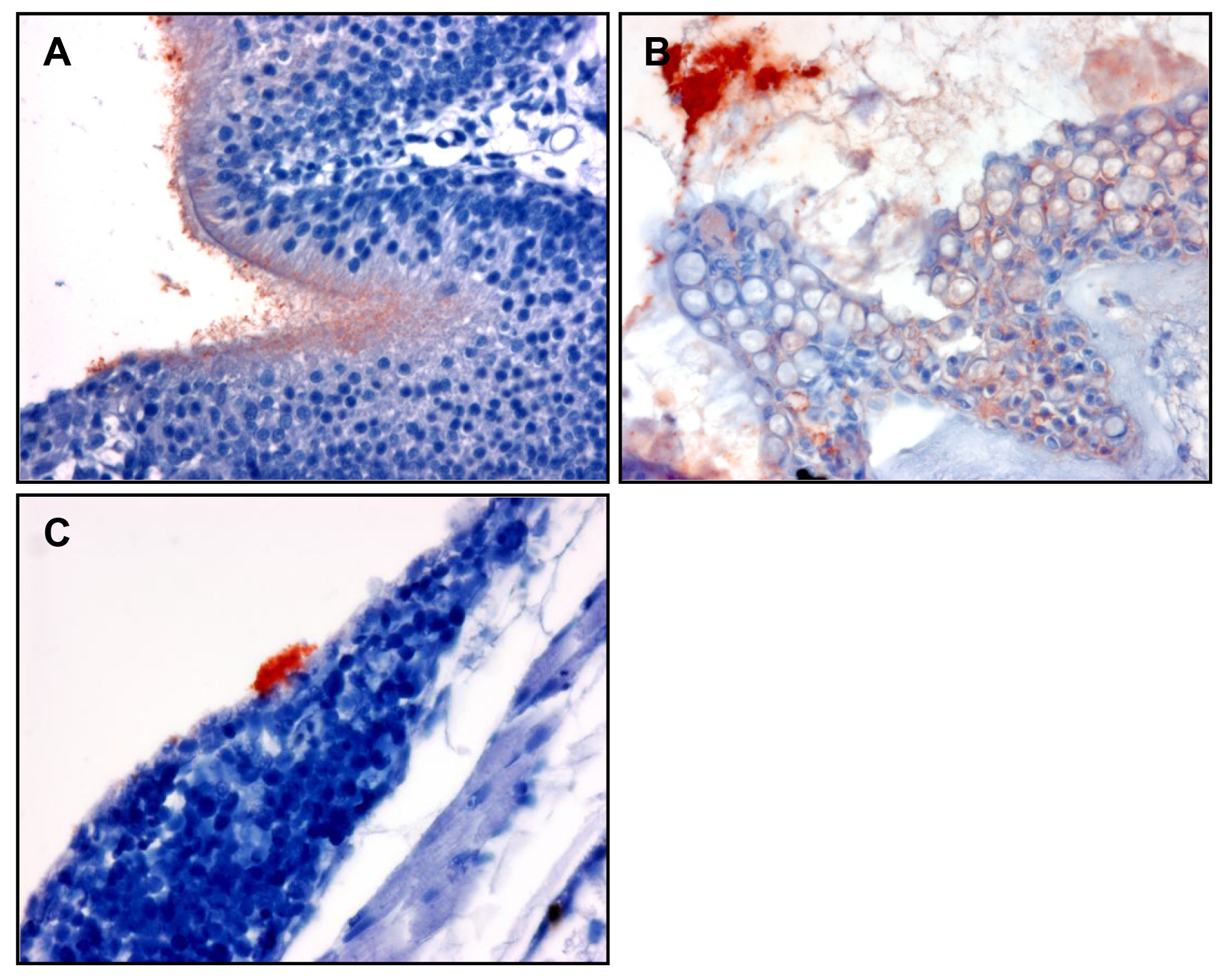

Supplement: S4 Fig — The sections of the GI tract fixed with Carnoy’s solution. (A) Olfactory bulb 30 seconds post vaccination, (B) mouth 30 minutes post vaccination, (C) thymus 30 seconds post vaccination were immunostained with anti-Y. ruckeri polyclonal antibody as described in materials and methods. (TIF) [file pone.0117263.s004.tif]
